# Malaria

**Published:** 1886-05

**Authors:** 


### MALARIA.

There is no more common term in use, to express the cause of a class of diseases incident to almost every section of our country, than that of *malaria*, and yet just what it is, and from what it proceeds has long been a mooted question, even among those who make the causes and cure of disease a life study.

It is generally conceeded that malaria is due to a poisonous state of the atmosphere superinduced by exhalations of decayed vegetable matter, in its exposure to the sun's rays in the warm seasons. However pleasant and inviting the warm spring days, they are not without their danger, especially to such as are devoted to an out door life. The heat of the sun dries up the putrefying vegetation that the winter has accumulated and fills the air with malaria. Spring-Fevers are very common and they are a very dangerous affliction. They arise from the enervated condition of the body; from the malarial influences of the atmosphere, and from the general change of the season. They show themselves in innumerable forms. The indications of their approach are a thirst for acids; yellow complexion; a weak circulation of the blood; unnatural condition of the skin; mysterious aching of the bones; a feeling of emptiness in the head; palpitation and irregularity of the heart; deficient or unnatural appetite; flatulence and constipation; a constant sense of weariness, with fluttering of the stomach, sinking sensations and dizziness. Any of these physical conditions must be treated promptly and thoroughly; the vitality must be restored, the system strengthened, for if they do not at once produce serious diseases, they are likely to lay the foundation of constitutional disorders.

There is scarcely a locality where malaria is not a leading cause of disease. In hot, moist climates it produces yellow fever; in temperate zones typhoid and intermittent fevers. It paralyses the liver and kidneys. These organs become filled with blood, producing enlarged liver, and chronic albuminaria, or Bright's disease of the kidneys, by interrupting the circulation of the blood. If the blood does not properly circulate, then it is not cleansed, and impure blood means decay and death. It has for years been a study with the most discerning physicians and scientists how best to counteract these destructive tendencies, or check them when they have once begun; severe exercise in the heat of the day and care less exposure to the chill night air, are conducive to malarial poisoning which takes the form of ague and other well known fevers. It is a mistake to allow the living or lodging rooms of your house to become damp. A very little fire in the grate will drive the enemy away, and a little lime or lemon juice and water, taken two or three times a day, will operate as a preventive and energize a torpid liver. There is wisdom in the old adage, "an ounce of preventive, is worth a pound of cure."

